# Mammalian Sperm Head Formation Involves Different Polarization of Two Novel LINC Complexes

**DOI:** 10.1371/journal.pone.0012072

**Published:** 2010-08-10

**Authors:** Eva Göb, Johannes Schmitt, Ricardo Benavente, Manfred Alsheimer

**Affiliations:** Department of Cell and Developmental Biology, Biocenter, University of Würzburg, Würzburg, Germany; Texas A&M University, United States of America

## Abstract

**Background:**

LINC complexes are nuclear envelope bridging protein structures formed by interaction of SUN and KASH proteins. They physically connect the nucleus with the peripheral cytoskeleton and are critically involved in a variety of dynamic processes, such as nuclear anchorage, movement and positioning and meiotic chromosome dynamics. Moreover, they are shown to be essential for maintaining nuclear shape.

**Findings:**

Based on detailed expression analysis and biochemical approaches, we show here that during mouse sperm development, a terminal cell differentiation process characterized by profound morphogenic restructuring, two novel distinctive LINC complexes are established. They consist either of spermiogenesis-specific Sun3 and Nesprin1 or Sun1η, a novel non-nuclear Sun1 isoform, and Nesprin3. We could find that these two LINC complexes specifically polarize to opposite spermatid poles likely linking to sperm-specific cytoskeletal structures. Although, as shown in co-transfection/immunoprecipitation experiments, SUN proteins appear to arbitrarily interact with various KASH partners, our study demonstrates that they actually are able to confine their binding to form distinct LINC complexes.

**Conclusions:**

Formation of the mammalian sperm head involves assembly and different polarization of two novel spermiogenesis-specific LINC complexes. Together, our findings suggest that theses LINC complexes connect the differentiating spermatid nucleus to surrounding cytoskeletal structures to enable its well-directed shaping and elongation, which in turn is a critical parameter for male fertility.

## Introduction

Anchorage and active positioning of the nucleus plays a fundamental role during diverse developmental processes such as fertilization, cell migration, establishment of polarity and is critical for differentiation of various cell types [1]–[3]. It essentially requires a direct interaction between the nucleus, in particular the nuclear envelope (NE), and the cytoskeleton. In this context, the so-called LINC complexes (linker of nucleoskeleton and cytoskeleton) gained more and more importance as they actually define the molecular basis to physically connect the nucleus to the peripheral cytoskeleton. LINC complexes are formed across the NE by the interaction of members of two transmembrane (TM) protein families: SUN and KASH domain proteins [4], [5].

SUN domain proteins are an evolutionary conserved family of inner nuclear membrane (INM) proteins that share a common C-terminal motif, the SUN (Sad1p/Unc84 homology) domain [6], [7]. The mammalian genome codes for at least five SUN proteins. The two major SUN proteins, Sun1 and Sun2, are widely expressed in different cell types [8], [9]. Sun3, Sun4 and Sun5 appear to have a more restricted, most likely testis-specific expression, but as yet remained rather uncharacterized [10]–[12]. SUN proteins are integral inner nuclear membrane proteins with an N-terminal nucleoplasmic region separated by a TM domain from the C-terminal part that extends into the perinuclear space (PNS) [8], [9]. Within the PNS, SUN proteins directly interact via their terminal SUN domain with the C-terminal KASH (Klarsicht/Anc1/Syne1 homology) domain of respective KASH protein partners [2], [13]. Mammals contain at least four KASH proteins, which are called nesprins [14]–[19]. Nesprins are outer nuclear membrane (ONM) proteins containing a long cytoplasmic N-terminus that has the ability to bind to the cytoskeleton [2]. Two of them, Nesprin1 and 2, are large actin-binding proteins [15], [20], whereas Nesprin3, a smaller molecule, binds to plectin that in turn links to the intermediate filament system and/or actin [18], [21]. Contrary, Nesprin4 is restricted to few cell types and binds to kinesin, a microtubule associated protein [19]. Tethering of nesprins to the ONM depends on the localization of Sun1 and Sun2 in the INM [4], [20], [21]. Since both Sun1 and Sun2 also interact with A-type lamins and other components of the INM (i.e. emerin) the SUN-KASH-interaction within the PNS forms a functional cross linkage of the nucleoskeleton and the cytoskeleton [4], [22].

Besides their primary function in connecting nucleoplasmic to cytoplasmic structures, LINC complexes are supposed to be directly involved in dynamic processes concerning anchorage and migration of nuclei (reviewed in [3]) but also positioning and movement of nuclear structures (i.e. meiotic telomeres) [23]–[24]. Mutations in either SUN or KASH proteins that result in defective LINC complex assembly lead to severe failures in nuclear migration, anchorage and organization [24]–[29]. Moreover, LINC complexes have been proposed to play a role in nuclear deformation and shaping [30]–[32].

Nuclear restructuring is very pronounced during spermiogenesis, a highly complex process which ensures the differentiation from haploid male germ cells into mature, fertilization competent spermatozoa. A most prominent feature in this process is the shaping of the sperm nucleus from spherical to elongated [33]. Nuclear remodeling during sperm head formation requires an elaborate cooperation of different cellular mechanisms that involve assembly of sperm-specific cytoskeletal structures, nuclear movement and chromatin compaction [34]–[38]. Thereby the initially round cell nuclei reshape to elongate. Nuclear elongation, however, is well-directed and leads to striking polarization concerning nuclear and cellular shape [39], [40]. Noteworthy, failures in sperm head shaping and formation in effect is a major cause of male infertility [41].

Recent studies indicate that elongation of the sperm nucleus involves an extensive modulation of the NE. This, in particular, concerns its general composition as well as the behavior of the single components. A central characteristic during sperm head elongation is the remarkable redistribution and polarization of a variety of NE components (e.g. lamins B1 and B3, Lap2 and lamin B receptor) [42]–[44]. At present, the functional significance of this redistribution is unclear. However, it was speculated that NE remodeling contributes to sperm-specific nuclear shaping and chromatin reorganization [43]–[45].

In a variety of cell types SUN and KASH proteins function as nucleo-cytoplasmic linkers that directly contribute to nuclear positioning and shaping. Therefore, LINC complexes are good candidates for playing a key role in nuclear deformation and elongation that is overt during mammalian sperm differentiation. In the present study, we investigated the expression of LINC complex components in the male postmeiotic germ line. We describe Sun3 as a novel spermiogenesis-specific member of the SUN protein family. In addition, we could find Sun1 showing an exceptional behavior in postmeiotic cells. We further were able to identify a novel testis-specific, non-nuclear Sun1 isoform which is exclusively expressed in spermatids and mature spermatozoa. Remarkably, analyzing the subcellular localization of SUN proteins and nesprins in differentiating spermatid nuclei we detected two specifically polarizing novel LINC complexes consisting of differentially interacting SUN and KASH pairs. Thus, we propose a model for a well-directed sperm head formation that involves the presence of two spermiogenesis-specific LINC complexes at opposite poles of the sperm head.

## Results

### Murine *Sunc1* codes for Sun3, a short testis-specific member of the SUN domain protein family

Previous studies described a short human SUN domain protein, SUN3, which was suggested to be expressed mainly in the testis [4], [5], [12]. However, up to the present the actual expression pattern as well as its subcellular distribution within the cells naturally expressing SUN3 is not known. To close this gap, we investigated SUN3 expression and localization in detail.

**Figure 1:**
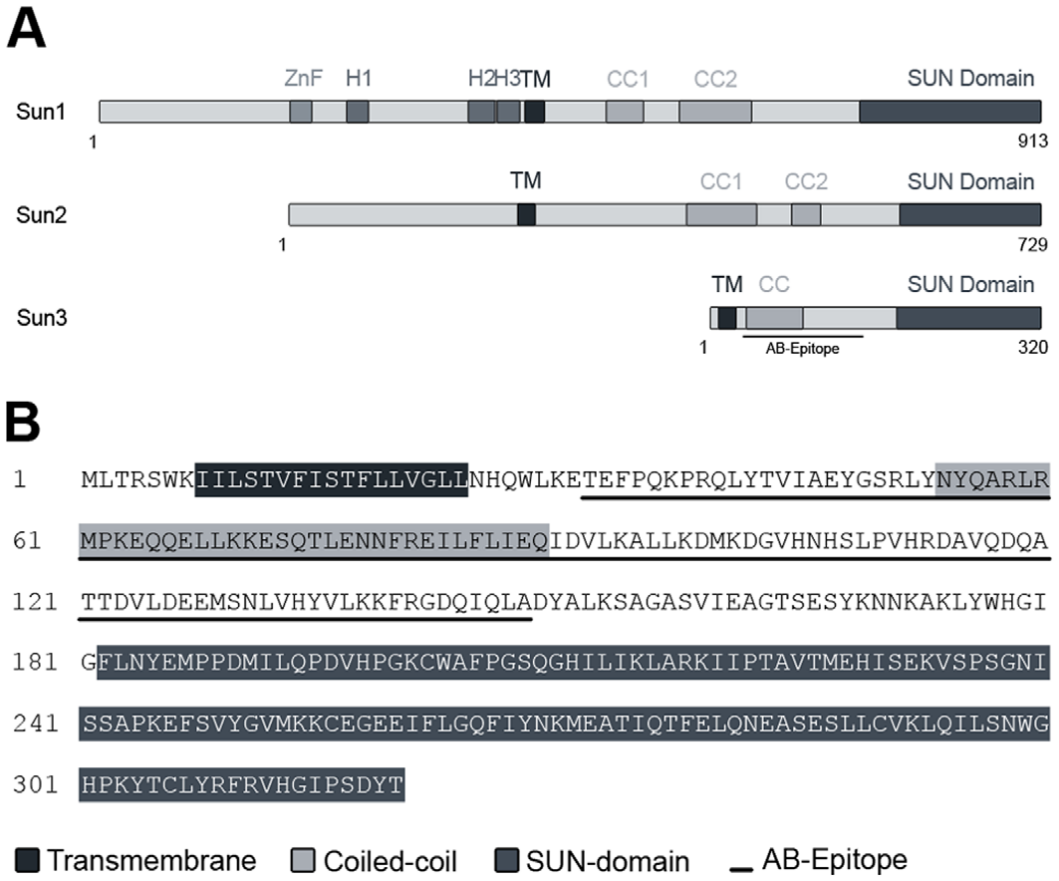
Sun3 is a novel member of the mammalian SUN protein family. (A) Schematic illustration of SUN domain proteins yet identified in mice. Relative positions of zinc finger (ZnF), hydrophobic regions (H1-H3), transmembrane domains (TM), coiled-coils (CC) and SUN domains are indicated. (B) Amino acid sequence of murine Sun3. Predicted structural motifs (TM, CC and SUN) are highlighted. The peptide sequence used for immunization is underlined.

To identify the murine homolog of human SUN3, we performed a TBLASTN search using human SUN3 peptide sequence (gb|AAH26189) as probe. This led to the identification of a cDNA derived from adult testis (gb|AK132922) encoded by the murine *Sunc1* gene (*Sad1 and UNC84 domain containing 1*; Gene ID: 194974). To isolate full-length murine Sun3 cDNA, we used an oligonucleotide corresponding to the 5’-untranslated region adjacent to the putative start codon and a 3’-oligonucleotide complementary to the end of the coding region. Using total testis RNA as template for RT-PCR, we were able to amplify a 985 bp cDNA with an open reading frame of 963 bp. The corresponding protein consists of 320 amino acid residues (Figure 1A) with a calculated molecular weight of approx. 36,800 and a pI of about 7.67. On the amino acid level murine Sun3 shows 71% identity (78% similarity) to the previously described putative human SUN3 [4].

PSORTII and SMART prediction of protein structural motifs revealed that Sun3 protein comprises distinct N- and C- terminal regions (Figure 1B). A short N- and a long C-terminal domain are separated by one predicted TM region thus characterizing Sun3 as a type II transmembrane protein, similar to other SUN proteins [12]. The presumptive lumenal C-terminus contains one putative coiled-coil region and a distal SUN domain representing the region of homology with the mammalian paralogues Sun1 and Sun2 (Figure 1B). With the exception of the SUN domain, Sun3 does not share any homology with other mammalian proteins. Thus, Sun3 is a typical member of the SUN protein family consistent with the basic protein conformation representative for SUN domain proteins.

**Figure 2:**
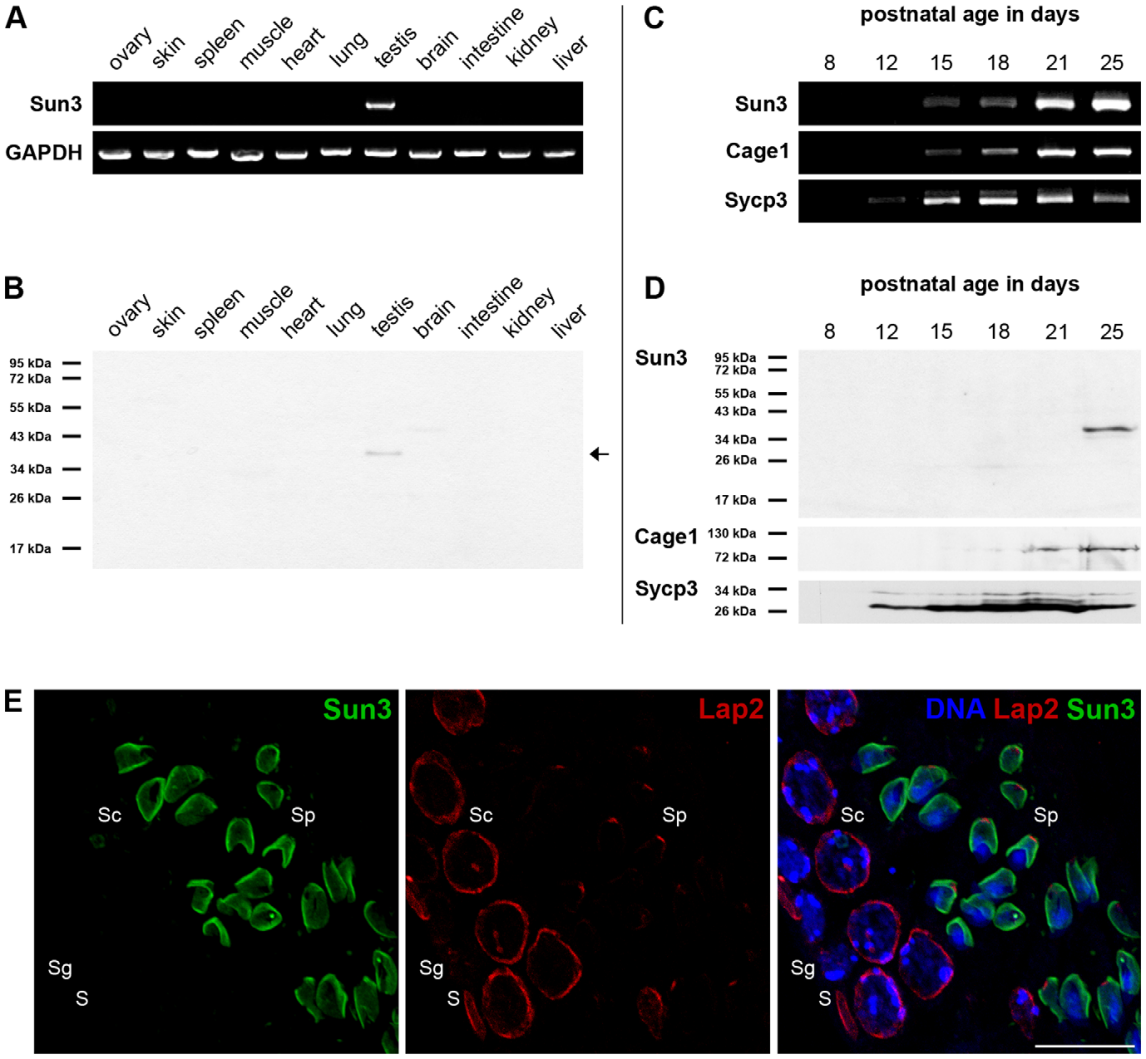
Sun3 is a testis specific protein showing a postmeiotic expression profile. (A) Total RNA was isolated from different tissues and used for RT-PCR. cDNA was amplified using Sun3-specific primers and a GAPDH-PCR served as control for RNA fidelity. (B) Samples from the same tissues were homogenized in SDS sample buffer and separated by SDS-PAGE (5×10^5^ cells; 30 µg protein/lane). Sun3 protein was detected with an affinity-purified anti-Sun3 antiserum (Arrow). (C) Total RNA was isolated from testicular cells from pubertal mice of increasing ages (8-25 days p.p.) and used for RT-PCR. cDNA was amplified using primers for Sun3, Cage1 (postmeiotic expression) and Sycp3 (meiotic expression). (D) To analyze stage-specific expression of protein Sun3 testes from pubertal mice were homogenized in SDS sample buffer and separated by SDS-PAGE (4×10^5^ cells/lane). Sun3 protein was detected with affinity-purified anti-Sun3 antiserum. For comparison pAbs against Lamin B3 and Cage1 were used. (E) Localization of Sun3 within seminiferous tubules was analyzed by indirect immunofluorescence microscopy. Testis paraffin sections of adult mice were stained using affinity-purified anti-Sun3 antiserum (green) and mAb 13d4 against LAP2 (red). DNA was labeled with 33258-Hoechst (blue). Sun3 was only detectable in spermatids (Sp) but completely absent from somatic cells (S), spermatogonia (Sg) and spermatocytes (Sc). Scale bar: 15 µm.

According to database entries the *Sunc1* gene appears mainly expressed in the testis as indicated by high representation as testis ESTs. To confirm the presumptive expression of Sun3 we first performed sensitive RT-PCR analysis on a broad range of different mouse tissues. Using sequence specific primers we could substantiate presence of Sun3 mRNA in the testis (Figure 2A). In contrast, we could not amplify Sun3 in any other somatic tissue or in the ovarian tissue sample. To verify tissue-specificity of Sun3 protein we performed western-blot analysis using an affinity-purified Sun3-antibody raised against a Sun3-specific peptide (Figure 1). The antibody recognized only one single protein band with the expected molecular mass of 37 kDa in the testis (Figure 2B). A corresponding signal, however, was clearly absent in all other tissues tested.

### Sun3 expression correlates with sperm head formation

We next performed a detailed analysis of Sun3 expression during spermatogenesis. Therefore, we used whole RNA fractions of 8- to 25- day-old mice that cover the first wave of spermatogenesis and consequently allow for exact timing of Sun3 appearance [42], [46], [47]. In 8- and 12-day old animals when testes only contain spermatogonia and spermatocytes, Sun3 message was not detectable (Figure 2C). However, with the appearance of first postmeiotic stages at days 15 and 18 postpartum (p.p.) the Sun3 message could be detected as a faint signal. As spermatogenesis progresses round and elongated spermatids begin to accumulate (i.e. days 21 and 25 p.p.) and, concomitantly, the Sun3 signal became stronger. For control, we performed RT-PCR analysis using primers for spermiogenesis-specific protein Cage1 (a component of the acrosome [48]) and meiotic protein Sycp3 (a structural component of the synaptonemal complex [49]). As expected for an acrosomal protein, Cage1 message first appeared in testes at day 15 p.p. with an increasing expression level thereafter which resembles the expression pattern of Sun3. By contrast, the Sycp3 message was detectable as early as day 12 p.p., when leptotene/zygotene spermatocytes are the most abundant cells within seminiferous tubules.

To confirm our results, we investigated the expression profile of Sun3 during spermatogenesis at the protein level as well. For this purpose, we prepared whole testicular suspensions from testes of pubertal mice at corresponding time points. Immunoblotting revealed that Sun3 was first detectable at day 25 p.p. when spermatids are most frequent within seminiferous tubules (Figure 2D). For control, we again used Sycp3 and Cage1 as marker for either meiotic or postmeiotic expression, respectively [50]. Summarizing, our observations clearly evidence that testis-specific Sun3 is expressed exclusively in postmeiotic stages of male germ cell development.

### Sun3 defines a novel LINC complex that colocalizes with the manchette

To study the distribution within the cells naturally expressing Sun3 (i.e. spermatids) we performed immunofluorescence microscopy on testis paraffin sections. In agreement with the previous results, somatic cells of the testis as well as spermatogonia and spermatocytes were negative for Sun3 (Figure 2E). In contrast, we could detect a strong Sun3-labeling at the NE of round and elongated spermatids (Figure 2E, Figure S1). Notably, Sun3 was not homogenously distributed within the nuclear periphery; it rather showed a highly polarized cap-like distribution.

**Figure 3:**
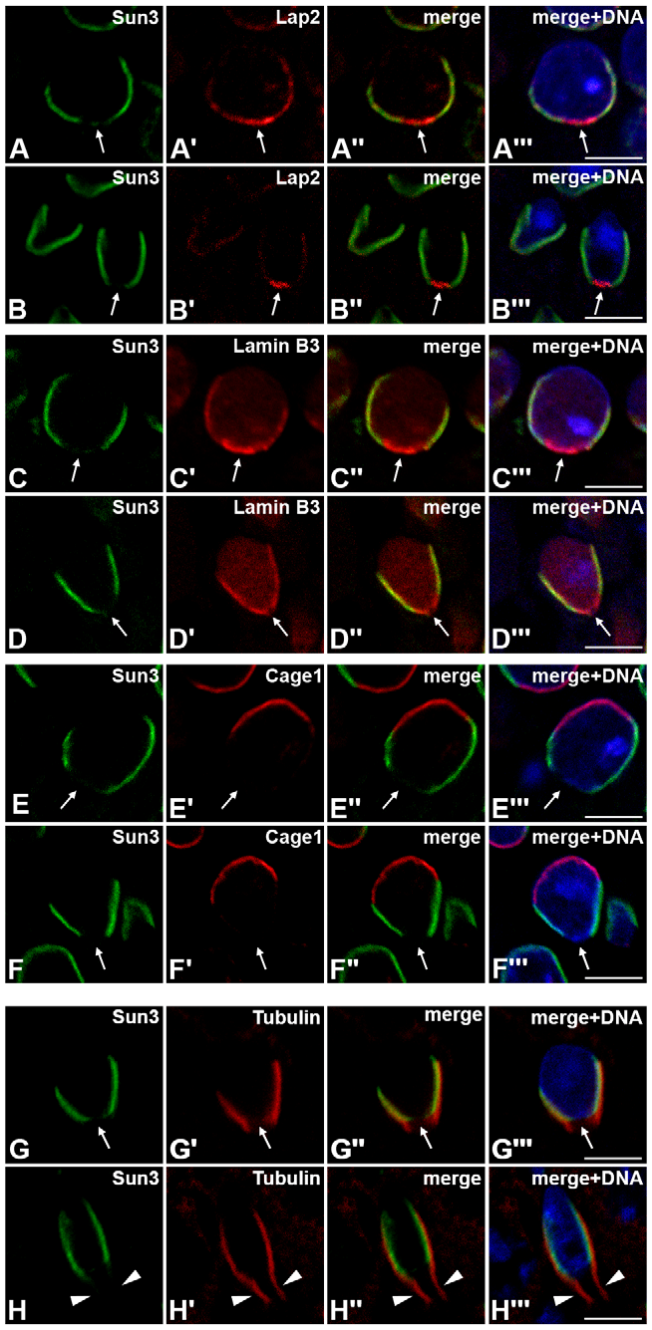
Sun3 polarizes to the posterior pole in differentiating spermatid nuclei colocalizing with manchette microtubules. (A–F) Distribution of Sun3 in round (A, C, E) and elongating (B, D, F) spermatids was investigated by indirect confocal immunofluorescence microscopy. Testis paraffin sections were co-stained using affinity-purified anti-Sun3 antiserum (A–F) and mAb 13d4 against LAP2 (A’, B’), pAb against Lamin B3 (C’, D’) or pAb against acrosomal protein Cage1 (E’, F’). Overlays are shown in A’’–F’’. DNA was labeled with 33258-Hoechst (A’’’–F’’’). Arrows indicate the fossa region. (G, H) Relation between the distribution of Sun3 and the localization of manchette microtubules in round (G) and elongating (H) spermatids was analyzed by indirect confocal immunofluorescence microscopy. Double-label immunofluorescence was performed on testis paraffin sections with affinity-purified anti-Sun3 antiserum (G, H) and mAbs against α- (H’) or β-tubulin (G’). Overlays are seen in G’’ and H’’. DNA was labeled with 33258-Hoechst (G’’’, H’’’). Arrows indicate the implantation fossa. Manchette microtubules extending to flagellar regions are denoted by arrowheads. Scale bars: 5 µm.

In double-label immunofluorescence experiments we compared the distribution of Sun3 with that of Lap2, another group of integral INM proteins [51] that gradually localize to the posterior pole of spermatid nuclei [43]. Both, Sun3 and Lap2 antibodies label the posterior pole of spermatid nuclei (Figure 3A,B). Similarly and consistent with previous findings, also lamin B3, a spermiogenesis-specific isoform of the *Lmnb2* gene [42], can be detected like Sun3 solely at the posterior pole (Figure 3C,D). However, Sun3 and the acrosome-specific protein Cage1 [48] localized to opposite poles of spermatid nuclei (Figure 3E,F).

Closer inspection of the spatial distribution of Sun3 and certain NE proteins revealed that the localization of Sun3 is unique and differs from that of the other proteins. Although Sun3 localizes like Lap2 and lamin B3 to the posterior pole, their local distribution within the NE shows remarkable differences; this implies that different NE proteins occupy distinguishable territories within the spermatid NE. At beginning of spermiogenesis lamin B3 and Lap2, both are distributed throughout the entire spermatid NE, but are locally enriched at the posterior half (Figure 3A,C). Sun3, by contrast, shows as early as cells are haploid complete posterior polarization, a distribution which is retained during complete spermiogenesis (Figure 2E, Figure 3). As sperm head formation progresses, lamin B3 and Lap2 gradually redistribute to congregate in a small region at the very posterior end of the nucleus which corresponds to the implantation fossa (i.e. the site where the flagellum anchors to the nucleus; Figure 3B,D). Although Sun3 colocalizes with lamin B3 and Lap2 in lateral regions of round spermatids, Sun3, however, remarkably appears completely excluded from the very posterior end of nuclei of round and elongated stages. Instead, Sun3 covers only lateral regions excluding the implantation fossa during entire sperm head elongation (Figure 3, Figure S2).

Absence of Sun3 at the very posterior pole could be confirmed by double-label immunofluorescences with antibodies to α- or β-tubulin. Both, α- and β-tubulin are the major structural components of the spermatid-specific manchette, a bundle of cytoplasmic microtubules which covers the posterior part of the nucleus throughout spermiogenesis [52] (see below). As shown in Figure 3G,H, Sun3 exclusively localizes to regions where microtubule bundles contact the NE. This fact is quite remarkable since it suggests a coherency between manchette formation and posterior localization of Sun3 and, moreover, provides a first hint for a putative involvement of Sun3 in nucleo-cytoskeletal linkage.

**Figure 4:**
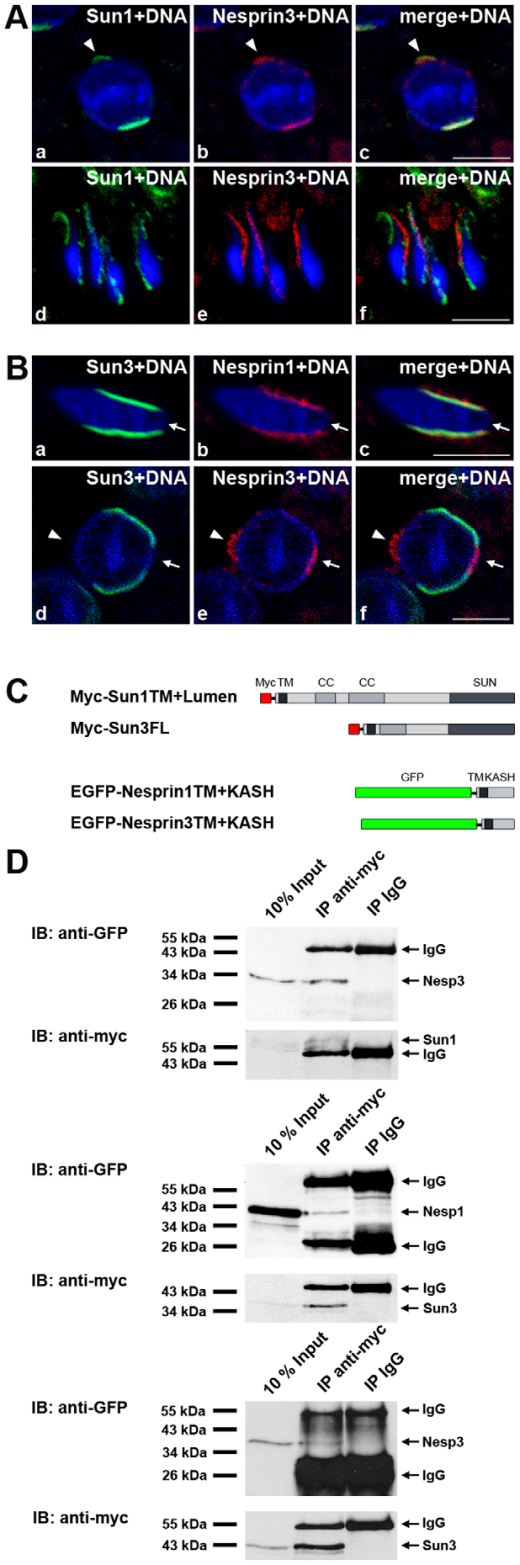
Appearance of two novel spermiogenesis-specific LINC complexes correlates with sperm head formation. (A, B) Distribution of Nesprins1 and 3 in spermatids. (A) Testis paraffin sections were co-stained using pAb against Nesprin3 together with affinity-purified anti-Sun1 antiserum. (B) Co-staining of testis paraffin sections using affinity-purified anti-Sun3 antiserum and pAb against Nesprin1 (b) or pAb against Nesprin3 (e). DNA was labeled with 33258-Hoechst. Overlays are seen in A, c, f and in B, c, f. Arrowheads denote the non-nuclear acrosome-associated Sun1/Nesprin3 localization. Arrows indicate the fossa region. Scale bars: 5 µm. (C) Schematic illustration of recombinant constructs used for co-transfection/immunoprecipitation experiments. (D) HeLa cells were transiently co-transfected with Myc-Sun1 and EGFP-Nesprin3 (top), Myc-Sun3 and EGFP-Nesprin1 (middle) or Myc-Sun3 and EGFP-Nesprin3 (bottom). Cells lysed in RIPA buffer were co-immunoprecipitated with anti-myc mAbs and immunoblotted with either anti-EGFP or anti-myc mAbs. For control, unspecific mouse IgG antibodies were used.

Following up this issue, we next asked whether Sun3 is part of a, thence, spermiogenesis-specific LINC complex. To this end, we performed immunofluorescence microscopy on testis paraffin sections using antibodies against yet known mammalian KASH proteins: Nesprins 1, 2, 3 and 4. Whereas Nesprins 2 and 4 were not detectable in any postmeiotic stage (not shown), Nesprins 1 and 3, both are actually present in spermatids as evidenced by immunofluorescence analysis (Figure 4A,B; see also below). Strikingly, Nesprin1 specifically localized to posterior regions of the NE during the nuclear elongation process and, moreover, entirely colocalized with Sun3 (Figure 4B). Notably, like Sun3, Nesprin1 was not detectable in the fossa region but was present only along lateral regions of developing spermatids.

These observations strongly point to a direct interaction between Sun3 and Nesprin1 in differentiating spermatids. Therefore, we next prepared co-transfection/immunoprecipitation experiments using truncated recombinant SUN and KASH protein constructs co-expressed in the mammalian HeLa cell culture system referring to the approaches described by Stewart-Hutchinson et al. [5]. We co-expressed Myc-tagged Sun3 together with the KASH domain of Nesprin1 or Nesprin3 coupled to an N-terminal EGFP. As control we used N-terminally Myc-tagged Sun1 lacking the nucleoplasmic domain together with an EGFP-Nesprin3 construct (Figure 4C). To analyze the interaction of these proteins in HeLa cells, immunoprecipitations were conducted with anti-myc antibodies and EGFP-nesprin proteins were detected using anti-EGFP antibodies. The ability of Sun3 to interact with KASH domain partners is displayed in Figure 4D showing that Myc-Sun3 co-immunoprecipitates with EGFP-Nesprin1 as well as EGFP-Nesprin3. This demonstrates an interaction of Sun3 and Nesprin1 in transfected mammalian cells, and moreover, substantiates our assumption based on the immunofluorescence experiments. As expected, under the same experimental conditions we were able to co-immunoprecipitated Myc-Sun1 with EGFP-Nesprin3 (Figure 4D) which is consistent with previous findings concerning a Sun1-Nesprin3 interaction [5].

Our results clearly demonstrate that Sun3 can participate in LINC complex formation through its interaction with Nesprin1. Moreover, Sun3 and Nesprin1, both jointly polarize to the posterior pole within the spermatid NE, a behaviour which is maintained throughout the sperm elongation process. Hence, our findings strongly indicate that Sun3 and Nesprin1 assemble a spermiogenesis-specific LINC complex which polarizes to the posterior pole, thereby facing sites where cytoplasmic manchette microtubules contact the NE.

### Sun1 is expressed during sperm head formation, but shows an exceptional behaviour

Because of their function in nuclear and chromosome dynamics and their suggested role in maintaining nuclear morphology [2], [30]–[32], [53], mammalian SUN proteins Sun1 and Sun2 are good candidates to have a major role in nuclear shaping during sperm head formation, too. Consistent with previous reports, we were unable to detect Sun2 in postmeiotic stages (not shown); Sun1, however, appears to be present during entire mammalian gametogenesis (Figure S3; see also [25]).

**Figure 5:**
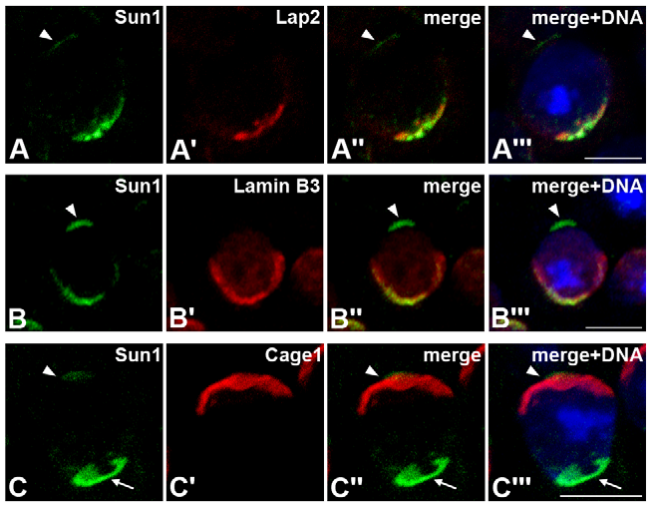
Sun1 displays a dual localization in early spermatid nuclei. Distribution of Sun1 in postmeiotic cells was investigated by indirect immunofluorescence microscopy. Testis paraffin sections were stained using affinity-purified anti-Sun1 antiserum (A-C). Same cells were co-stained for mAb 13d4 against LAP2 (A’), pAb against lamin B3 (B’) or pAb against acrosomal protein Cage1 (C’). Overlays are seen in A’’-C’’. DNA was labeled with 33258-Hoechst (A’’’-C’’’). Arrowheads indicate non-nuclear acrosome-associated localization of Sun1 (A-C). Arrow denotes the implanation fossa. Scale bars: 5 µm.

In order to unravel the spatial distribution of Sun1 in spermatids, we performed double-label immunofluorescence analysis using affinity purified antibodies to Sun1 together with antibodies to Lap2, Lamin B3 and Cage1. In round spermatids Sun1 mainly localizes to the posterior pole of the nucleus, a localization which was already visible in early stages, and, in principle, resembles that of other NE proteins including Sun3. However, contrary to lamin B3 and Lap2 but conform to Sun3, Sun1 is excluded from the implantation fossa (Figure 5, Movie S1). Moreover, in addition to the posterior polarization, we found an exceptional and unexpected localization of Sun1 at the anterior pole of the sperm head (Figure 5). Even more striking was the finding that at the anterior pole Sun1 localized outside the nucleus which became quite evident by double staining against Sun1 and the acrosomal marker Cage1. The Sun1 signal appeared anterior, but right on top of the acrosome indicating that it is part of the anterior acrosomal membrane system (Figure 5, Movie S1; see also below). Notably, acrosome-associated localization strictly differs from all other yet described NE proteins expressed during mammalian spermiogenesis, i.e. Lap2, LBR, lamins B3 and B1 and Sun3 (Figure 3, Figure 5).

Remarkably, as spermiogenesis progresses, we found a striking change in Sun1 distribution in that it gradually disappeared from the posterior pole, whereas the signal at the anterior pole of the sperm head became progressively stronger (Figure 4A, Figure 5B, Figure S3). In elongated spermatids, however, Sun1 was only detectable at anterior regions covering most parts of the acrosome but was completely absent form the posterior pole (Figure 4A d-f, Figure S3).

These findings clearly evidence that Sun1 in fact is expressed in all spermatogenic stages. In spermatids, Sun1 initially displays a dual localization; however, in the course of differentiation it gradually accumulates at the anterior pole.

### Sperm head formation involves expression of Sun1η, a novel spermiogenesis-specific Sun1 isoform

**Figure 6:**
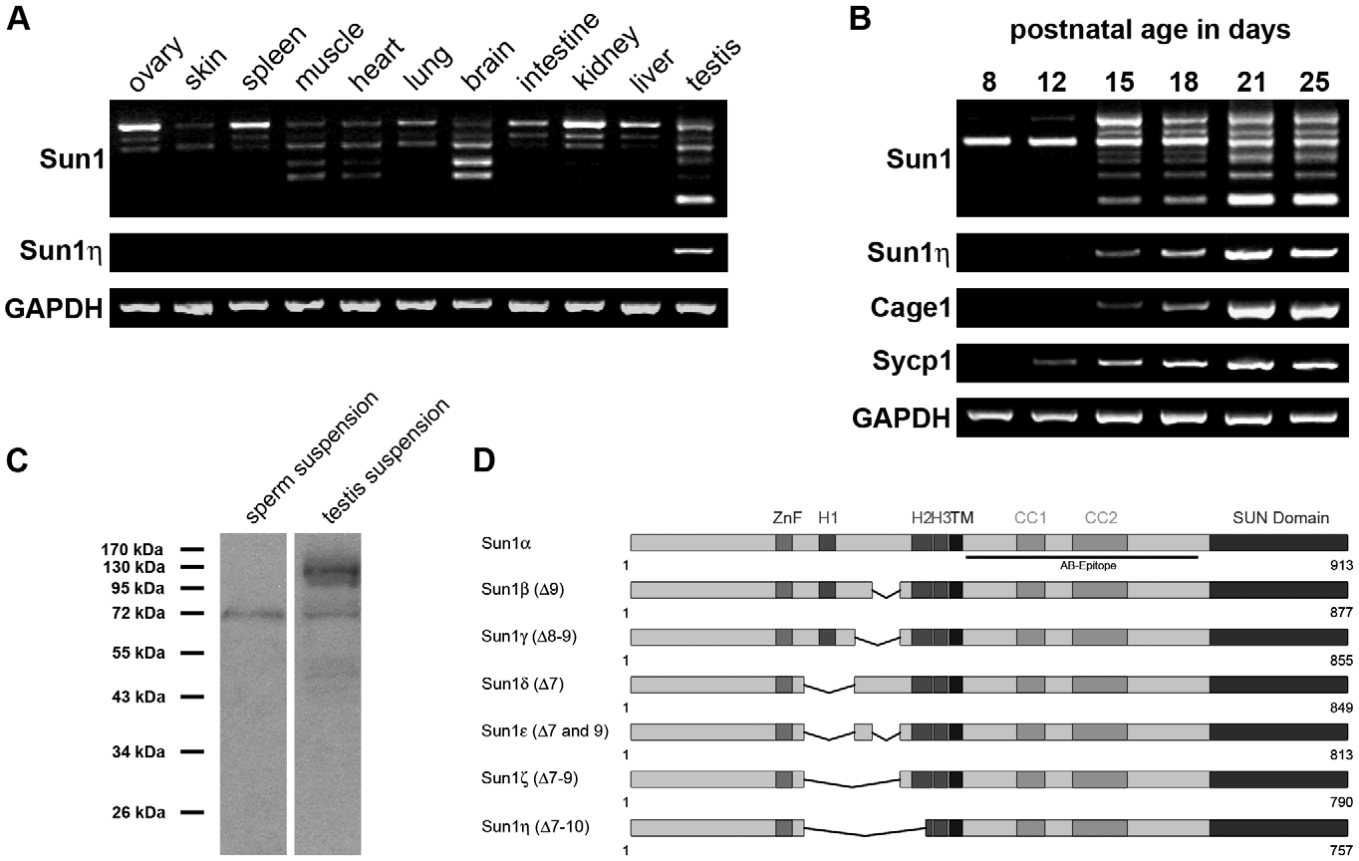
Novel Sun1 isoform Sun1η is testis-specific and exclusively expressed in postmeiotic stages of sperm development. (A) Presence of N-terminal Sun1 variants in different mouse tissues was analyzed by RT-PCR. To amplify predicted N-terminal Sun1 isoforms primers annealing in exon 6 and 11 were used. To verify testis-specificity and expression profile of Sun1η a primer pair that specifically amplifies Sun1η was applied. GAPDH served as control for RNA fidelity. (B) Appearance of Sun1η during mouse sperm differentiation was determined by RT-PCR. Total RNA from pubertal mice of increasing ages (8-25 days p.p.) served as template. cDNA was amplified using the same primers as in (A). Cage1 (postmeiotic expression), Sycp1 (meiotic expression) and GAPDH (RNA fidelity) were used for control. (C) To analyze the expression of protein Sun1η, suspensions from fractioned sperm heads (1×10^6^ cells) and whole testicular suspensions (5×10^5^ cells) were homogenized in SDS sample buffer and separated by SDS-PAGE. Affinity-purified anti-Sun1 antiserum was used for detection of respective Sun1 isoforms. (D) Schematic representation of the Sun1 isoforms yet identified in different mouse tissues. Epitope used for antibody production is indicated.

The anterior polarization and rather unexpected non-nuclear localization lead us to investigated Sun1 expression with more detail. Recently, it was shown that the N-terminal, nucleoplasmic domain of Sun1 is critical for NE retention [12], [22]. Therefore we speculated that non-nuclear Sun1 localization in spermatids could be due to expression of a distinct Sun1 splice variant that lacks sequences required for NE retention. To clarify this, we performed RT-PCR analysis to amplify N-terminal regions of all Sun1 isoforms described so far [12] (for primer details see Table S1). We used total RNA from different tissues in order to monitor the general expression profile of Sun1 isoforms that vary within their nucleoplasmic domain. We were able to identify three to seven distinguishable Sun1 messages depending on the tissue tested (Figure 6A). Sequencing of all these isoforms revealed that the different Sun1 transcripts lacked specifically one or more exons in the part coding for the nucleoplasmic domain (Figure 6D). Remarkably, in our approach we could identify a novel so far undescribed transcript lacking exons seven to ten, that we called Sun1η. Expression of this isoform appears to be restricted to the testis since it could not be amplified in any other tissue (Figure 6A). To verify testis-specificity of Sun1η, we used an additional primer set specific for the Sun1η isoform. Thereby, we could detect only one single message in the testis tissue sample confirming that expression of Sun1η is indeed restricted to the testis.

To examine the expression profile of Sun1η during mammalian spermatogenesis, we next performed RT-PCR on total RNA of pubertal testes [46], [47]. Using the same primers as above, Sun1η was only present in postmeiotic stages of spermatogenesis (Figure 6B). In testes that only contain spermatogonia and spermatocytes; i.e. 8- and 12-day-old animals, Sun1η was not detectable at all. However, at days 15 and 18 p.p., when first spermatids appear within seminiferous tubules, a faint Sun1η specific signal could be amplified. With accumulation of round and elongated spermatids (days 21–25 p.p.) the Sun1η signal became significantly stronger. Using Sycp1 and Cage1 as meiotic or postmeiotic marker, respectively, we could find that expression of Sun1η differs from meiotic Sycp1, but resembles that of spermiogenesis-specific Cage1 (Figure 6B) and, more important, that of Sun3 (Figure 2C,D) demonstrating that Sun1η expression is restricted to spermiogenesis.

To obtain information about Sun1η protein, we isolated mature spermatozoa obtained from epididymes of adult mice, separated sperm heads from tails and prepared protein suspensions from the sperm head fraction. Suspensions from isolated sperm heads were compared with whole testicular protein suspensions in immunoblotting experiments using specific antibodies against Sun1 (Figure 6C). Remarkably, the antibodies recognized only one single protein band in the sperm head fraction with a molecular mass of 72 kDa which is consistent with the calculated mass of Sun1η. This protein band was also detectable in whole testis suspension, albeit and as expected, higher molecular mass bands corresponding to full-length Sun1 or other Sun1 isoforms were present, too. Remarkably, these bands could not be detected in mature spermatozoa indicating that Sun1η is the only Sun1 isoform expressed at the end of sperm differentiation (Figure 6C).

Our results obtained by RT-PCR and immunoblotting strongly suggest that the newly identified Sun1 isoform, Sun1η, is testis-specific and selectively expressed during spermiogenesis. Remarkably, missing exon seven to ten, Sun1η lacks sequences proposed to be required for nuclear retention (see also [12], [22]).

### Sun1η expression correlates with the appearance of an anterior non-nuclear LINC complex

LINC complexes are present in many, if not all cell types; however, all yet identified LINC complexes specifically localize to the NE. As shown above, at the anterior pole of spermatids Sun1 localizes in a non-nuclear manner (Figure 5), a distribution which strongly correlates with the expression of Sun1η. To investigate if non-nuclear Sun1 forms a distinct LINC complex, we analyzed presence and behavior of respective KASH binding partners. As mentioned above, we detected both, Nesprin1 and 3 at the NE of spermatids, with Nesprin1 entirely colocalizing with Sun3 (Figure 4B). In contrast, Nesprin3 showed a quite different localization and its behavior remarkably resembled that of Sun1 (Figure 4A). Like Sun1, Nesprin3 was initially found at the posterior pole of round and early elongating spermatids and also at the anterior side of the sperm head (Figure 4A a–c). In late elongated spermatids, however, both proteins were exclusively found at the anterior pole (Figure 4A d–f). Notably, Nesprin3 and Sun3 although being able to interact with each other (Figure 4D) did not colocalize at all, but rather appeared to be mutually exclusive (Figure 4B d–f). Since the C-terminal SUN domain of Sun1 is capable to directly interact with the KASH domain of Nesprin3 (Figure 4D; [12]) it can be assumed that during sperm head formation at the anterior pole Sun1 (most likely Sun1η; see above) and Nesprin3 together form a functional, however non-nuclear LINC complex.

In summary, during sperm head formation SUN and KASH domain proteins appear to form distinct LINC complexes that, strikingly, polarize to opposite poles with Sun1η/Nesprin3 localizing to the anterior and Sun3/Nesprin1 to the posterior side.

## Discussion

**Figure 7:**
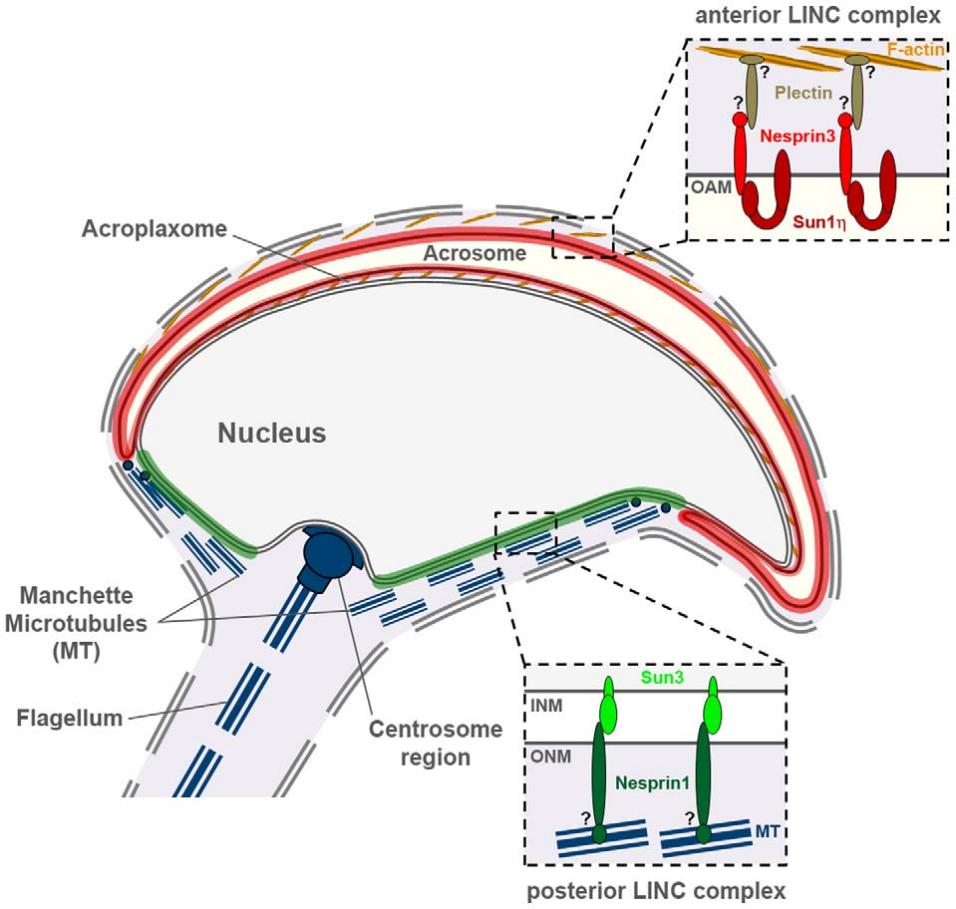
Model illustrating LINC complex polarization during sperm head formation. Two novel spermiogenesis-specific LINC complexes polarize to opposite nuclear poles where they appear to connect the nucleus/acrosome to surrounding manchette microtubules (MT) and F-actin, respectively. The anterior polarizing non-nuclear LINC complex (red) consists of Sun1η and Nesprin3, both mainly located within the outer acrosomal membrane (OAM). Interacting with plectin Sun1η/Nesprin3 LINC complexes could anchor to anterior actin filaments present at membranous junction of spermatids and Sertoli cells. The posterior polarizing LINC complex (green) is formed by Sun3 and Nesprin1. Interaction of Nesprin1 with microtubules (i.e. via dynein/dynactin and/or kinesin II) would enable the linkage of Sun3/Nesprin1 LINC complexes to the posterior manchette. Thus, both poles of differentiating spermatid nuclei would be connected to cytoskeletal elements by two distinctive LINC complexes.

In eukaryotes, cellular and nuclear shaping is crucial for multiple events including cell migration, division and differentiation of diverse cell types. Also spermiogenesis, the differentiation of motile, fertilization competent spermatozoa, is a matter of fundamental morphogenic restructuring. Initially round nuclei are deliberately deformed to achieve their compact and elongated structure. The molecular mechanisms responsible for this morphological remodeling are largely unknown yet far. However, the cytoskeleton appears to be of fundamental importance since spermatids establish specialized cytoskeletal structures that are suggested to play a role in sperm-specific nuclear shaping; either in providing forces to actively elongate nuclei or in positioning nuclei to be shaped by exogenous forces [34], [38], [52]. While spermatids differentiate these cytoskeletal elements become visible at opposite regions of spermatid nuclei. At the anterior side develops the acroplaxome a thin cytoplasmic sheet mainly built-up by F-actin between elongating nucleus and acrosome whereas the posterior half of the nucleus is covered by the spermatid-specific manchette a calyx-like structure formed of bundles of microtubules [33]–[35] (Figure 7). Remarkably, both structures tightly appose to differentiating nuclei almost anchoring directly to the NE. Taking these facts to account, consequently, the question arises how these cytoskeletal elements and forces could be connected to the elongating nucleus. Here we show for the first time that LINC complexes are also established in male postmeiotic germ cells. We also provide compelling evidence that these complexes are good candidates for anchoring spermatid head components to surrounding cytoskeletal structures, thus enabling deliberate sperm head shaping.

### Two novel LINC complexes, Sun1η/Nesprin3 and Sun3/Nesprin1, during sperm head formation

In the present study we demonstrated that differentiating spermatids express two novel LINC complexes: Sun1η/Nesprin3 and Sun3/Nesprin1. Our immunocytochemical and biochemical experiments clearly evidence that mammalian Sun1η and Sun3 actually are testis-specific proteins with an expression profile exactly correlating with postmeiotic sperm differentiation. Furthermore, we characterize Sun3 as a Nesprin1 interacting protein that can participate in LINC complex formation thus enlarging the potential of mammalian cells to establish multiple LINC complex variants.

Notably, our immunofluorescence data indicate that in spermatids these two novel LINC complexes are not homogenously distributed in the NE as in somatic cells. However, they accumulate specifically in distinct regions of the elongating sperm head: LINC complexes consisting of Sun3/Nesprin1 polarize to the posterior pole of spermatid nuclei whereas LINC complexes formed by Sun1η/Nesprin3 show an atypical non-nuclear localization at the anterior pole (Figure 7).

Directed polarization of NE components per se reflects a general feature of mammalian spermiogenesis: profound modulation of the entire NE which is suggested to influence nuclear elongation [42], [43], [45]. However, our experiments uncovered remarkable divergences between single NE components concerning their individual polarization. Particularly Sun3 differed strikingly from all other NE proteins analyzed thus far. NE proteins (including Sun1), like nuclear pore complexes, progressively redistribute to the posterior pole before they apparently disappear as spermiogenesis progresses ([42]–[44], [54] and this study). However, Sun3 although resembling in principle the posterior polarization of the other NE components, differs due to its one-to-one colocalization with manchette microtubules over the time and its absence from the fossa region. Therefore, we suggest that during different stages of sperm differentiation subsets of NE proteins occupy distinct and distinguishable NE territories thus enabling fundamentally new roles for NE proteins, especially for LINC complex components.

Our results strongly suggest that LINC complexes established in the male postmeiotic germline are distinctly formed by either Sun1η and Nesprin3 or Sun3 and Nesprin1. Interestingly, Sun1η and Nesprin1 both are present at the same time in the same cell but, although having the competence to bind each other ([5]; unpublished data), they actually do not interact, at least in the given cellular context. Similarly, via its SUN domain Sun3 has the potential to directly interact with the KASH domain of Nesprin3, but this alternative to form a LINC complex appears excluded during spermiogenesis. Hence, our data show for the first time that, even though diverse SUN and KASH populations might be present within the same cell, they do not generally share their LINC complex partners. Rather, they can distinguish between the partners to form accurately defined LINC complexes which could be assigned for distinct tasks. This appears at least to be true for postmeiotic germ cells: two different but well-defined LINC complexes polarize to opposing poles and hence link to different cytoskeletal structures (Figure 7; see below). However, how SUN proteins can take a decision to which KASH partner they actually bind and how this differential binding is regulated, remains an interesting and important quest for the future.

Previous studies have reported that structural integrity of LINC complexes as well as the general amount of their components are critical parameters for maintaining nuclear shape. In particular, Olins and colleagues [30] documented that granulocytes, a class of highly deformable white blood cells having exceptional shaped nuclei (i.e. highly lobulated), are characterized by a remarkable paucity of LINC complex components. This specific modulation during granulocyte differentiation could directly affect nucleo-cytoskeletal linkage and in turn is suggested to be an evolutionary adaptation to facilitate nuclear and cellular deformability [30]. Evidence for a direct influence of SUN domain proteins on nuclear morphology came from a study which showed that in *Dictyostelium* the expression of an N-terminally truncated Sun1 led to its mislocalization and thence to severe nuclear deformations [31]. Likewise, depletion of or mutations in nesprins have dramatic consequences on general nuclear architecture and shape [32], [55] and affected nuclei show serious deformations [56].

Thus, the amounts as well as the formation of functional LINC complexes are in fact critical for nuclear morphology and shape. However, given that not only decreased levels of LINC complex components or their mislocalization can alter nuclear shape but also target-oriented redistribution of SUN and KASH proteins, it appears quite conceivable that LINC complexes are also critical for mediating sperm head formation.

### The Sun3/Nesprin1 LINC complex at the posterior sperm head pole

The presence of Sun1η/Nesprin3 anterior as well as Sun3/Nesprin1 posterior remarkably resembles the distribution of spermatid-specific cytoskeletal structures. Our data obtained by immunofluorescence microscopy reveal a coherency between the manchette and the posterior Sun3/Nesprin1 polarization. Interestingly, Russell and collegues [34] previously reported that rod-like elements of constant length were linking the innermost microtubules of the manchette to the outer surface of the NE and, even more, to the inner leaflet of the NE across the PNS. Furthermore, they and also others [38], [52] share the opinion that these links between the manchette and the NE provide a structural basis for the transmission of forces to elongate nuclei. Sun3/Nesprin1 LINC complexes could finely reflect these NE-manchette-connecting filaments. Moreover, Nesprin1 has potential binding ability to KIF3B, a subunit of kinesin II [57], and the dynein/dynactin complex [58]. Therefore its interaction with manchette microtubules via kinesin II and/or dynein/dynactin would be conceivable (Figure 7). However, Kierszenbaum and Tres [35] demonstrated that F-actin, to which Nesprin1 is rather related, is also present in the manchette. Therefore, actin could be the primary cytoskeletal element to which Sun3/Nesprin1 LINC complexes are connected as well.

### The Sun1η/Nesprin3 LINC complex at the anterior sperm head pole

Though Sun1/Nesprin3 initially polarize to the posterior pole, a distinct proportion is also found at the anterior pole in early round spermatids. With progressing differentiation anterior localization becomes more and more prominent while the posterior proportion disappears. Most striking, at the anterior pole Sun1/Nesprin3 does not localize to the NE but appears outside the nucleus likely being part of the acrosomal membrane system. This behavior, however, is quite remarkable since Sun1 as an INM protein has never been observed before to localize purposefully outside the NE.

What might be the reason and the functional significance of this exceptional Sun1 localization? As shown in our study, the appearance of the non-nuclear LINC complex closely correlates with the expression of a new, spermiogenesis-specific Sun1 isoform, Sun1η, which lacks exons seven to ten. Loss of these exons, however, implies that Sun1η does not contain hydrophobic sequences H1 and H2 in part. Recently, Liu and colleges [12] reported that these hydrophobic sequences are required for INM targeting and nuclear retention. Since the NE is profoundly restructured during spermiogenesis including redistribution and disappearance of certain components (see above) it is plausible that the long Sun1 variant is initially recruited to the posterior pole but successively disappears in the course of differentiation. Sun1η however, due to absence of N-terminal sequences required for efficient INM retention [12] could fail to associate with nuclear interaction partners and, as a consequence, relocates to the non-nuclear acrosomal membrane system. Accordingly, by direct interaction of their respective SUN or KASH domains (this study and [5]), Sun1η has the competence to recruit Nesprin3 to form a functional, however non-nuclear LINC complex at the anterior pole of the sperm head.

In somatic cells Nesprin3 apparently links the nucleus to the intermediate filament system via its interaction with plectin [18], [21], a cytoskeletal cross-inker protein which is suggested to influence cytoskeletal organization and mechanical strengthening of cells [59]–[61]. Plectin for its part is further able to interact with actin [62] which in spermatids is enriched within the acroplaxome and at specialized membranous junctions of spermatids and Sertoli cells at the anterior sperm surface [35], [63], [64]. Notably, both anterior occurring structures appear, like the posterior manchette to be involved in shaping of the sperm head. Interestingly, plectin was also reported to be concentrated at those junctions between Sertoli cells and elongating spermatids potentially linking to adjacent actin filaments [65], [66]. For that reason, we propose a model in which Sun1η/Nesprin3 LINC complexes anchor via plectin to these actin containing structures (Figure 7). This linkage in turn would provide a structural basis for the location of the Sun1η/Nesprin3 LINC complex to the anterior pole and, in addition, via closely connected NE-acrosomal-plasma membrane systems to the outer surface of the acrosome (Figure 7). In this way spermatid nuclei could become tethered to anterior cytoskeletal structures by the spermiogenesis-specific LINC complex presented here.

Taken together we conclude that mammalian sperm head formation involves two novel opposed polarizing LINC complexes which connect differentiating nuclei to surrounding sperm-specific cytoskeletal elements. Thus, our findings provide a first hint how spermatid nuclei could be anchored to cytoskeletal structures at opposite sides of the sperm head to enable its gradual shaping and elongation.

## Materials and Methods

### Ethics Statement

All animal care and experimental protocols were conducted in accordance with the guidelines provided by the German Animal Welfare Act (German Ministry of Agriculture, Health and Economic Cooperation). Animal housing and breeding was approved by the regulatory agency of the city of Würzburg (Reference ABD/OA/Tr; according to §11/1 No. 1 of the German Animal Welfare Act). Personnel from our laboratory carried out all aspects of the mouse work under strict guidelines to insure careful, consistent and ethical handling of mice.

### Animals and tissue preparation

Tissues used in this study were obtained from C57BL/6 and CD1 mice. Somatic tissues as well as testis tissue samples from adult and pubertal mice (8–25 days p.p.) were processed for either RNA isolation or protein analysis as previously described [48]. Mature spermatozoa were obtained from epididymes of adult mice and sperm heads were fractioned as described in Longo et al. [67].

### cDNA cloning and generation of plasmid constructs

The putative human SUN3 protein sequence (GenBank accession number AAH26189) served as query to search for murine homologous sequences in the NCBI database by the TBLASTN algorithm. This search identified one cDNA clone of 1180 bp (GenBank accession number AK132922) containing the complete coding region of murine Sun3. For cloning mouse Sun3 full-length cDNA, total RNA was isolated from a testicular suspension of an adult mouse using TriFAST™ (Peqlab Biotechnology) according to manufacturer’s protocol. Reverse transcription was performed on 1 µg of total RNA with oligo(dT) primer and M-MLV reverse transcriptase (Promega). Based on the database sequence a 5’ oligonucleotide, corresponding to a sequence upstream of the start codon (Sun3-5’; primers and PCR-conditions are denoted in Table S1) and a 3’ oligonucleotide, complementary to the 3’ end of the coding region (Sun3-3’), were selected for PCR amplification. The obtained Sun3 cDNA was cloned into pCR®2.1-TOPO® vector (Invitrogen) and sequenced according to standard protocols. cDNA encoding full-length murine Sun1 (GenBank accession number BC047928) was obtained from RZPD (Deutsches Ressourcenzentrum für Genomforschung GmbH). For antibody production, expression vectors coding for Sun3 and Sun1 antigens fused to C-terminal His–tags were generated. A Sun3 fragment coding for amino acids 31–148 was amplified by PCR from full-length Sun3 (Sun3AB-5’Nde; Sun3AB-3’Xho), digested with the respective enzymes and cloned into *Nde*I/*Xho*I sites of pET-21a(+) (Novagen). Similarly, a Sun1 fragment coding for amino acids 427-722 was amplified from full-length Sun1 (Sun1AB-5’Nde; Sun1AB-3’Eco) and cloned into *Nde*I*/EcoR*I sites of pET-21a(+) [68]. To generate Myc-tagged constructs for immunoprecipitation, PCR fragments of Sun3 coding for the full-length protein (Sun3FL: Sun3-5’Eco; Sun3-3’Sal) and Sun1 coding for amino acids 501–913 (Sun1TM+Lum: Sun1IP-5’Eco; Sun1IP-3’Sal) were cloned into pCMV-Myc vector (Clontech Laboratories). To create GFP-tagged KASH domains either of Nesprin1 or Nesprin3, Nesprin1TM+KASH (74 C-terminal amino acids of murine Nesprin1) and Nesprin3TM+KASH (67 C-terminal amino acids of Nesprin3) fragments were amplified by RT-PCR from NIH/3T3 fibroblasts. PCR products were cloned into the *Sma*I site of pEGFP-C2 (Clontech).

### Expression analysis by RT-PCR

Expression profiles of Sun1 and Sun3 were analyzed by RT-PCR using total RNA, isolated from testes of adult mice as well as from testicular suspensions of pubertal mice (8–25 days). Reverse transcription was performed on 1 µg of total RNA as described above. To monitor Sun1 RNA expression that includes predicted N-terminal variable isoforms [4], [12] we used a 5’-oligonucleotide corresponding to a sequence in exon 6 (Sun1Exp-5’) and a 3’-oligonucleotide, complementary to the sequence in exon 11 (Sun1Exp-3’). To verify testis-specificity and expression profile of Sun1η a second primer pair that specifically amplifies Sun1η was applied (Sun1ATG-5’; Sun1η-3’). Sun3 expression was analyzed by using primers Sun3AB-5’Nde and Sun3AB-3’Xho.

### Antibodies

For the generation of specific antibodies against Sun1 and Sun3, His-tagged Sun1 and Sun3 fusion constructs were expressed in *E. coli* Rosetta™ (Novagen), purified under denatured conditions on Ni-NTA agarose resin (Qiagen) and used for immunizing of guinea pigs (Seqlab, Göttingen, Germany). Obtained sera were affinity purified on HiTrap™ NHS-activated HP Columns coupled with the respective antigens (GE Healthcare) as described by the manufacturer, and tested for specificity by immunofluorescence microscopy and immunoblotting. Further antibodies used in this study were: affinity purified rabbit anti-lamin B3 [42]; affinity purified rabbit anti-Cage1 [48]; monoclonal antibody 13d4 against LAP2 [43]; affinity purified rabbit anti-SYCP3 [50]; and a rabbit polyclonal antibody against Nesprin3 (kindly provided by Dr. Arnoud Sonnenberg, The Netherlands Cancer Inst., Amsterdam, The Netherlands) [18]. Rabbit anti-SYNE1 (HPA019113) was purchased from Atlas Antibodies. Monoclonal antibodies against α-Tubulin (T5168) and β-Tubulin (T4026) were obtained from Sigma-Aldrich. Monoclonal mouse anti-GFP (B-2) was purchased from Santa Cruz Biotechnology. Mouse monoclonal anti-myc antibody (R950-25) was obtained from Invitrogen. Secondary antibodies coupled to Cy2 and Texas Red as well as peroxidase-conjugated secondary antibodies were obtained from Dianova.

### Immunocytochemistry

For immunostaining, testes of adult mice were fixed for 3 hours at room temperature in PBS containing 1% paraformaldehyde, embedded in paraffin and sectioned (sections 3–10 µm). Paraffin removal and antigen retrieval was performed as previously described [48]. After permeabilization with PBS containing 0.1% Triton X-100 for 10 minutes sections were blocked for 1 hour with PBT (0.15% BSA, 0.1% Tween 20 in PBS). Then sections were subjected for 1 hour to respective primary antibodies. After washing twice in PBS, specimens were incubated with appropriate secondary antibodies conjugated to Cy2 or Texas Red. DNA was stained with Hoechst 33258 (Hoechst). Digital images were taken by confocal microscopy with a Leica TCS-SP2 AOBS confocal laser scanning microscope (63x/1.40 HCX PL APO oil-immersion objective, pinhole at 1 P AU; Leica). All images shown in this study are maximum 2D projections calculated either of three or of all sequenced single sections. Image data were processed using Adobe Photoshop and Amira® Software.

### Cell culture and transfection

HeLa cells were grown in 10% bovine growth serum, 1% L-glutamine, 1% penicillin/streptomycin with DMEM (Invitrogen). Cells were cotransfected with the respective pEGFP- and pCMV-Myc- expression vectors using Effectene™ transfection reagent (Qiagen) according to manufacturer’s instructions and incubated for 24 hours at 37°C and 5% CO_2_ content.

### Co-immunoprecipitation

Co-immunoprecipitation was performed as described by Stewart-Hutchinson et al. [5]. HeLa cells grown to confluency on 35-mm dishes were harvested 24 hours post transfection with the respective constructs and lysed with 500 µl of radio-immunoprecipitation assay (RIPA) lysis buffer (1% Triton X-100, 0.5% sodiumdesoxycholate, 0.1% SDS, 1 mM β-glycerophosphate, 1 mM Na_3_VO_4_, 1 mM EDTA, 1 mM EGTA, in PBS, protease inhibitor mixture). Lysates were cleared by centrifugation at 15.000*g* in a microfuge at 4°C for 15 minutes and the supernatant was divided into 250 µl aliquots. Each aliquot was then incubated with either 1.5 µg monoclonal mouse anti-myc antibody (Invitrogen) or mouse IgG unspecific antibody (Dianova). After overnight incubation at 4°C with continuous shaking, immune complexes were pulled down with Dynabeads® Protein G (Invitrogen): 20 µl of the magnetic beads were added, incubated for 2 hours at 4°C, harvested by magnet and washed three times with 250 µl RIPA buffer. Finally the beads were resuspended in 2xSDS-sample buffer (120 mM Tris/HCl, 10% SDS, 20% Glycerol, 20% 2-mercaptoethanol, bromphenol blue, pH 6.8), heated to 95°C for 15 minutes and resolved by SDS-PAGE.

### SDS-PAGE and Western blotting

Protein samples resulting from immunoprecipitation experiment, samples of different mouse tissues as well as testicular cells from mice of different ages (8–25 days) were resuspended in 2xSDS-sample buffer and heated to 95°C for 15 minutes. Proteins were separated by SDS-PAGE and transferred to nitrocellulose membranes. Membranes were blocked over night at 4°C in TBST buffer (10 mM Tris/HCl, 150 mM NaCl, 0.1% Tween 20, pH 7.4) containing 10% milk powder and incubated for 1 hour at room temperature with respective primary antibody in blocking solution. After washing with TBST buffer, membranes were incubated with appropriate secondary antibodies coupled to peroxidase. Signals were detected using the Western Lightning® Plus-ECL, Enhanced Chemiluminescence Substrate (Perkin Elmer).

## Supporting Information

Table S1 Oligonucleotides used in the study.
(0.06 MB DOC)

Figure S1 Sun3 expression during mammalian spermiogenesis. Localization of Sun3 within seminiferous tubules was analyzed by indirect immunofluorescence microscopy. Testis paraffin sections of adult mice were stained using an affinity-purified anti-Sun3 antiserum (green). DNA was labeled with 33258-Hoechst. Sun3 is detectable in round (A) as well as in elongated (B) spermatids but not in other spermatogenic cell types. Scale bar, 50 µm.
(1.22 MB TIF)

Figure S2 Posterior polarization of Sun3 in round and elongated spermatids. Localization of Sun3 in round (A and B) and elongated (C and D) spermatids was investigated by indirect immunofluorescence microscopy followed by 3D reconstruction. Testis paraffin sections of adult mice were stained using an affinity-purified anti-Sun3 antiserum. DNA was labeled with 33258-Hoechst (A'-D'). Arrows indicate the region of the implanation fossa. Images of sequenced single sections were taken by confocal laser scanning microscopy and 3D reconstruction of the scans was calculated using Amira® Software. Scale bars, 5 µm.
(0.46 MB TIF)

Figure S3 Sun1 expression during mammalian spermatogenesis. Localization of Sun1 within seminiferous tubules was analyzed by indirect immunofluorescence microscopy. Testis paraffin sections of adult mice were stained using an affinity-purified anti-Sun1 antiserum (green). DNA was labeled with 33258-Hoechst (blue). Sun1 is present in spermatocytes (Sc; punctured distribution corresponding to meiotic telomeres), in round (rSp; cap like distribution at the posterior pole) and in elongated spermatids (eSp; cap like distribution at the anterior pole). Scale bar, 15 µm.
(0.78 MB TIF)

Movie S1 Localization of Sun1 and acrosomal protein Cage1 in a 3D reconstructed round spermatid. Testis paraffin section of an adult mouse was co-stained for Sun1 (green) and acrosomal protein Cage1 (red). DNA was labeled with 33258-Hoechst (blue). Images of sequenced single sections were taken by confocal laser scanning microscopy and reconstruction of the scans was calculated using Amira® Software.
(1.58 MB MPG)
