# Supplementary material for: Mammalian Sperm Head Formation Involves Different Polarization of Two Novel LINC Complexes
Source: PLoS One. 2010 Aug 10;5(8):e12072. doi: 10.1371/journal.pone.0012072 (PMC2919408; doi:10.1371/journal.pone.0012072)
Supplement: Table S1 — Oligonucleotides used in the study. (0.06 MB DOC) [file pone.0012072.s001.doc]

|  | **Primer** | **Sequence** | **Annealing** | **Cycles** |
| --- | --- | --- | --- | --- |
| 1 | Sun3-5’ | 5’-CCCTTTGGTCAACTAACTTGCA-3’ | 55°C | 25 |
| 2 | Sun3-3’ | 5’-CTAAGTGTAATCACTGGGGATGC-3’ |
| 3 | Sun1-AB-5’Nde | 5’-CATATGGGTGCTGGTGTCTCCCTGTG-3’ | 55°C | 25 |
| 4 | Sun1AB-3’Eco | 5’-GAATTCTGCGCTTCTGTGATTCCTGAA-3’ |
| 5 | Sun3AB-5’Nde | 5’-CATATGACAGAGTTTCCTCAAAAACCAGG-3’ | 55°C | 25 |
| 6 | Sun3AB-3’Xho | 5’-CTCGAGAGCCAACTGTATTTGGTCCCCT-3’ |
| 7 | Sun1IP-5’Eco | 5’-GAATTCATATTTGCAAGGTTTTTGTCTTG-3’ | 60°C | 30 |
| 8 | Sun1IP-3’Sal | 5’-GTCGACCTACTGGATGGGCTGTCC-3’ |
| 9 | Sun3-5’Eco | 5’-GAATTCCAATGTTAACTCGATCATGGAAG-3’ | 60°C | 30 |
| 10 | Sun3-3’Sal | 5’-GTCGACCTAAGTGTAATCACTGGGGATG-3’ |
| 11 | Nesp1IP-5’ | 5’-TCCGATTCCTCCCGTTCTGACC-3’ | 62°C | 30 |
| 12 | Nesp1IP-3’ | 5’-TCTCCTCTTACTGCCTGCACTTGTGTG-3’ |
| 13 | Nesp3IP-5’ | 5’-CGGAGGCAGAAGCGGTGGAG-3’ | 60°C | 30 |
| 14 | Nesp3IP-3’ | 5’-CAGAGGCAGCATTTGGTCTAAGGCT-3’ |
| 15 | Sun1Exp-5’ | 5’-CAGCAATGGATACACTTGCCGTG-3’ | 60°C | 30 |
| 16 | Sun1Exp-3’ | 5’-CCAGAAGGTTCCCGAGGCTG-3’ |
| 17 | Sun1ATG-5’ | 5’-ATGGACTTTTCTCGGCTGCAC-3’ | 66°C | 30 |
| 18 | Sun1η-3’ | 5’-GCTGCCTTCCGTGGTTTGAG-3’ |
| 19 | GAPDH-5’ | 5’-GGGCCCACTTGAAGGGTGGAGC-3’ | 58°C | 25 |
| 20 | GAPDH-3’ | 5’-GTCAGATCCACGACGGACACATTGG-3’ |
| 21 | Cage1-5’ | 5’-GCGACTCTTAAAGAGTTAATTGCTAGTG-3’ | 60°C | 25 |
| 22 | Cage1-3’ | 5’-GGCACCATAAAGAATGTTCTATTTCCTTGGATCC-3’ |
| 23 | Sycp3-5’ | 5’-GGCTTCGTCAGATGCTTCGAG-3’ | 52°C | 30 |
| 24 | Sycp3-3’ | 5’-GACTCATCAGAATAACATGGATTGAAG-3’ |
| 25 | Sycp1-5’ | 5’-GGATCCGCATCTTTACTGGAATCAC-3’ | 60°C | 30 |
| 26 | Sycp1-3’ | 5’-CCCGGGTTAAGAAAATAACTTTTCTGC-3’ |
