# Supplementary figures and images for: Mammalian Sperm Head Formation Involves Different Polarization of Two Novel LINC Complexes
Source: PLoS One. 2010 Aug 10;5(8):e12072. doi: 10.1371/journal.pone.0012072 (PMC2919408; doi:10.1371/journal.pone.0012072)

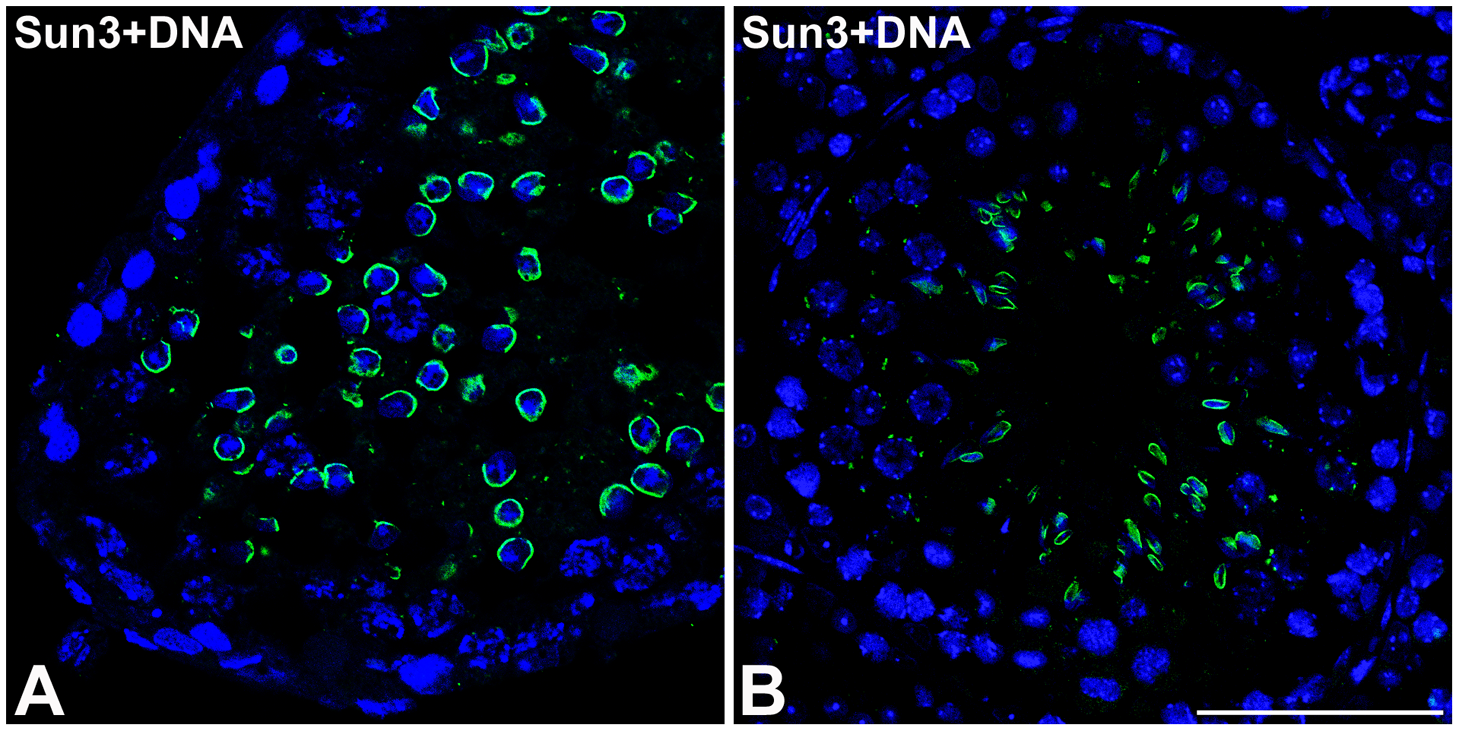

Supplement: Figure S1 — Sun3 expression during mammalian spermiogenesis. Localization of Sun3 within seminiferous tubules was analyzed by indirect immunofluorescence microscopy. Testis paraffin sections of adult mice were stained using an affinity-purified anti-Sun3 antiserum (green). DNA was labeled with 33258-Hoechst. Sun3 is detectable in round (A) as well as in elongated (B) spermatids but not in other spermatogenic cell types. Scale bar, 50 µm. (1.22 MB TIF) [file pone.0012072.s002.tif]

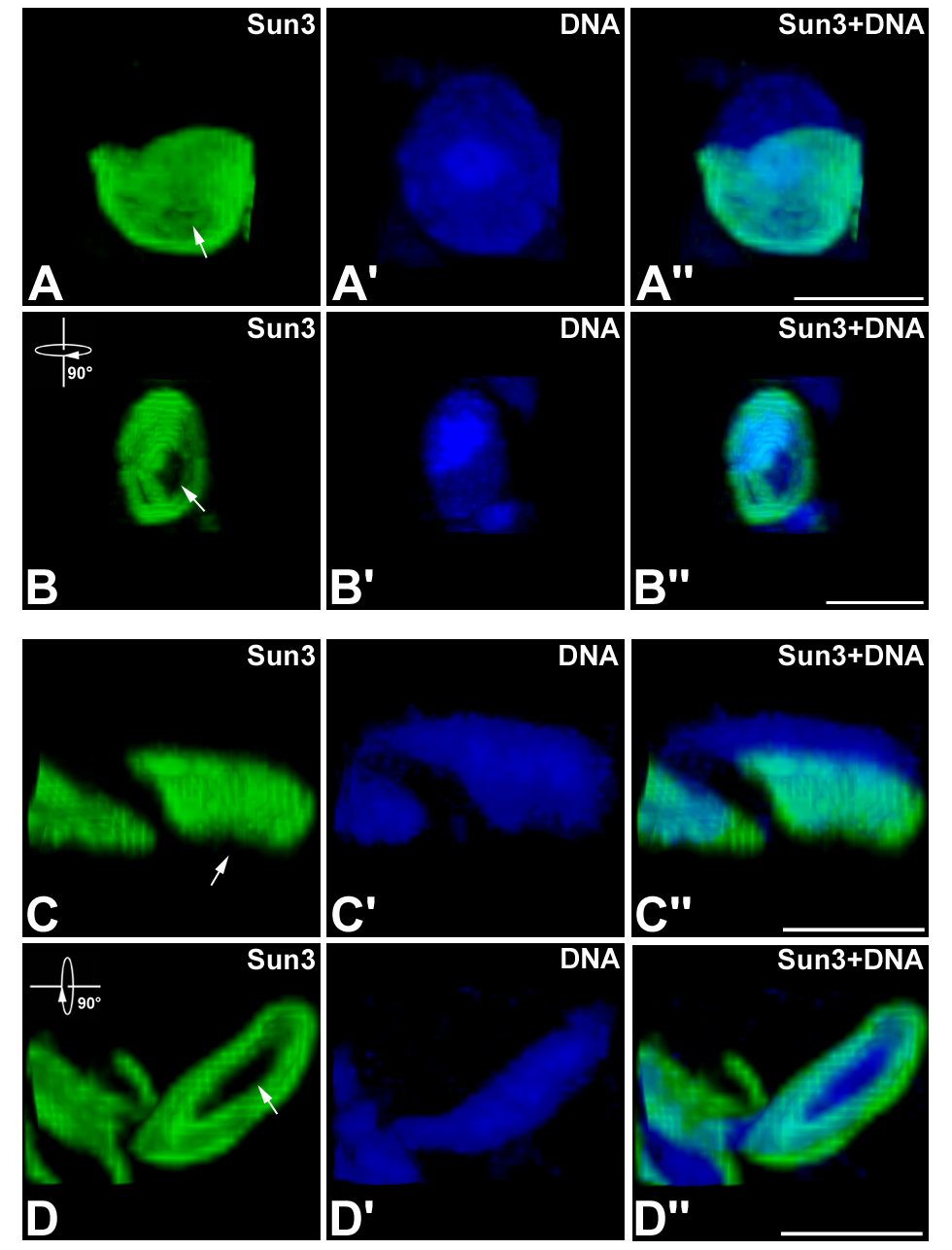

Supplement: Figure S2 — Posterior polarization of Sun3 in round and elongated spermatids. Localization of Sun3 in round (A and B) and elongated (C and D) spermatids was investigated by indirect immunofluorescence microscopy followed by 3D reconstruction. Testis paraffin sections of adult mice were stained using an affinity-purified anti-Sun3 antiserum. DNA was labeled with 33258-Hoechst (A'-D'). Arrows indicate the region of the implanation fossa. Images of sequenced single sections were taken by confocal laser scanning microscopy and 3D reconstruction of the scans was calculated using Amira® Software. Scale bars, 5 µm. (0.46 MB TIF) [file pone.0012072.s003.tif]

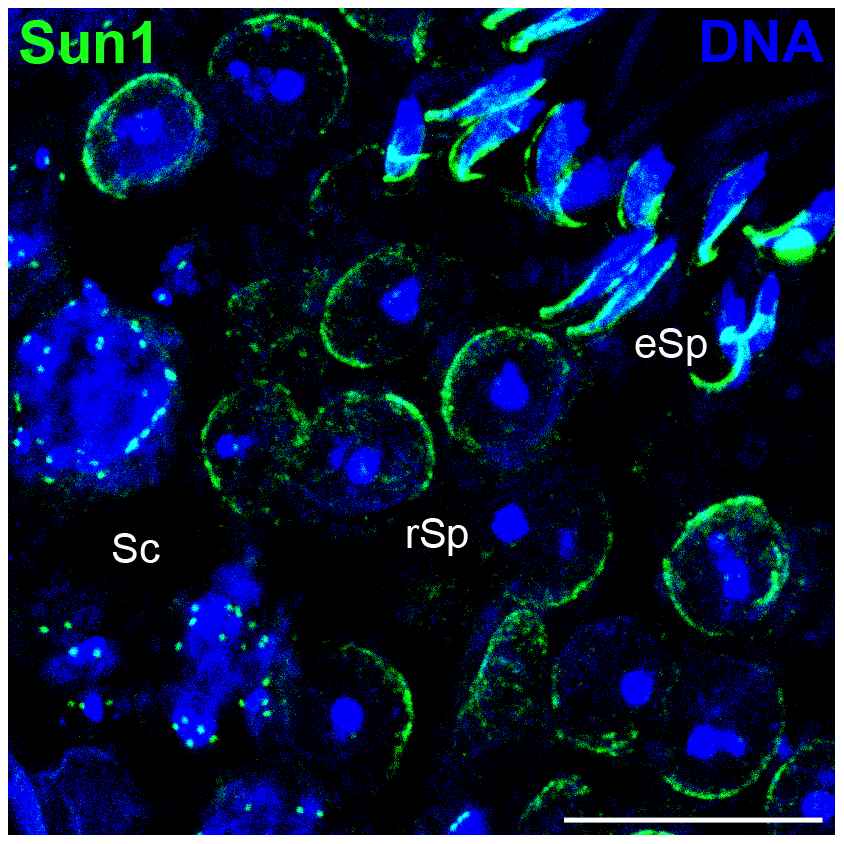

Supplement: Figure S3 — Sun1 expression during mammalian spermatogenesis. Localization of Sun1 within seminiferous tubules was analyzed by indirect immunofluorescence microscopy. Testis paraffin sections of adult mice were stained using an affinity-purified anti-Sun1 antiserum (green). DNA was labeled with 33258-Hoechst (blue). Sun1 is present in spermatocytes (Sc; punctured distribution corresponding to meiotic telomeres), in round (rSp; cap like distribution at the posterior pole) and in elongated spermatids (eSp; cap like distribution at the anterior pole). Scale bar, 15 µm. (0.78 MB TIF) [file pone.0012072.s004.tif]
